# Periodontal Disease and Risk of Bladder Cancer: A Meta-Analysis of 298476 Participants

**DOI:** 10.3389/fphys.2018.00979

**Published:** 2018-07-23

**Authors:** Wen-Zhong Xie, Ying-Hui Jin, Wei-Dong Leng, Xing-Huan Wang, Xian-Tao Zeng, BPSC investigators

**Affiliations:** ^1^Department of Stomatology, Kaifeng Stomatological Hospital, Kaifeng Central Hospital, Kaifeng, China; ^2^Center for Evidence-Based and Translational Medicine, Zhongnan Hospital of Wuhan University, Wuhan, China; ^3^Department of Stomatology, Taihe Hospital, Hubei University of Medicine, Shiyan, China; ^4^Department of Urology, Zhongnan Hospital of Wuhan University, Wuhan, China

**Keywords:** Periodontal diseases, periodontitis, urinary bladder neoplasms, cohort studies, meta-analysis

## Abstract

**Objective:** It has been reported that the periodontal disease is linked to a number of malignant tumors such as lung cancer and pancreatic cancer. In this study, we aimed to investigate the association of periodontal disease with risk of bladder cancer by a meta-analysis.

**Methods:** PubMed, Scopus, ScienceDirect, and Chinese National Knowledge Infrastructure (CNKI) were searched for eligible publications up to December 15, 2017. Cohort and nested case-control studies on the association between periodontal disease and risk of bladder cancer were included. After study selection and data extraction, pooled hazard ratios (HRs) and their 95% confidence intervals (95%CIs) were calculated using a fixed-effect inverse-variance model. All analyses were performed using the RevMan 5.3 software.

**Results:** Finally, five cohort studies were identified and included in this meta-analysis, involving 1,104 bladder cancer cases of 298,476 participants. Summary estimates based on adjusted data showed that periodontal disease was not significantly associated with the risk of bladder cancer (*HR* = 1.09, 95% CI = 0.95–1.25, *I*^2^ = 0%). A similar result was also observed after cumulative, subgroup and sensitivity analyses.

**Conclusions:** Current evidence from cohort studies suggests that patients with periodontal disease may not be at an increased risk of developing bladder cancer.

## Introduction

According to the 2015 Global Burden of Disease (GBD) study, the cumulative burden of oral conditions including untreated dental caries, severe chronic periodontitis and total tooth loss had dramatically increased between 1990 and 2015, as accounting for a 64% increase in disability-adjusted life years (DALYs); and the incident cases in 2015 were 616 million (Kassebaum et al., 2017), which posed a huge public health challenge to policy makers. In China, from 1990 to 2013, the periodontal disease (PD) standardized DALYs rate had increased slightly from 24.7 to 25.7, based on the data from 2013 GBD study (Zhang et al., 2017). Moreover, it is indicated in accumulating evidence that PD associated with the increased risk of various systemic diseases, such as cardiovascular disease (Zeng et al., 2017), asthma (Moraschini et al., 2018), overweight/obesity (Martens et al., 2017), inflammatory bowel disease (Papageorgiou et al., 2017), systemic lupus erythematosus (Rutter-Locher et al., 2017), diabetes mellitus (Ziukaite et al., 2018), carotid atherosclerosis (Zeng et al., 2016a), and erectile dysfunction (Cheraghi and Doosti-Irani, 2017). Furthermore, some researches also suggested that PD may be involved in the pathogenesis of malignant tumors (Sadighi Shamami and Amini, 2011; Michaud et al., 2017). The associations of PD with lung cancer (Zeng et al., 2016b), gastric cancer (Yin et al., 2016), head and neck cancer (Zeng et al., 2013), and pancreatic cancer (Maisonneuve et al., 2017) have been proved in multiple meta-analyses (Zeng X. et al., 2015), which further deepen the understanding of the relationship between PD and cancer risk. Obviously, the prevention and treatment of PD have been a focus of medicine, especially in China. Bladder cancer (BC), the 11th most commonly diagnosed cancer, is another serious disease worldwide (Ferlay et al., 2008). The morbidity and mortality rates of BC vary across countries due to the differences in risk factors, detection and diagnostic practices, and availability of treatments (Bosetti et al., 2011; Burger et al., 2013). Current evidence indicates that smoking is an independent risk factor for BC in both men and women (Freedman et al., 2011).

As we have observed, PD may increase the risk of several cancers (Sadighi Shamami and Amini, 2011; Zeng et al., 2013, 2016b; Yin et al., 2016; Maisonneuve et al., 2017; Michaud et al., 2017), and tobacco smoking is an important common risk factor of PD and BC (Waziry et al., 2017). In addition, in 1863 Virchow hypothesized that cancer might originate from lesions by chronic inflammation, and subsequent evidence demonstrated that inflammation was a critical component of cancer progression (Coussens and Werb, 2002). PD is a chronic inflammatory disease affecting the supporting structures of the teeth, which is induced by pathogenic bacteria (Pihlstrom et al., 2005; Yan et al., 2014; Zeng X. T. et al., 2015). Hence, it is easy to understand that oral cavity and bladder can be a reservoir of oral microbiome and pathogens. Since a number of observational studies investigating the risk of BC in patients with PD were available, we performed this meta-analysis (Zeng X. et al., 2015) of all eligible observational studies to estimate the association between PD and risk of BC. This research was reported following the Preferred Reporting Items for Systematic Reviews and Meta-Analyses (PRISMA) statement (Moher et al., 2009).

## Materials and methods

### Eligibility criteria

According to the “PICOS” (population/disease, exposure, control, outcome, and study design), the studies meeting each of the following criteria were included in the meta-analysis: (1) the study (S) adopted a prospective cohort, retrospective cohort, or nest case-control design and provided full text; (2) the disease (P) was BC, the exposure (I) of interest was PD and the control (C) was free of PD; (3) the outcome (O) of interest was incident BC (including incidence rate and cancer mortality); (4) adjusted risk ratios (RRs), incidence density ratios, or hazard ratios (HRs) with their associated 95% confidence intervals (95% CIs), and the adjustment for confounding factors were reported. If more than one reports came from the same cohort, we analyzed them and then chose the best one.

### Search strategy

We searched PubMed, Scopus, ScienceDirect and Chinese National Knowledge Infrastructure (CNKI) to identify all relevant studies published in English or Chinese up to December 15, 2017 using the following keywords: periodontal disease, periodontitis, and cancer. Reference lists of included studies as well as the newest reviews and editorials were also screened for additional studies.

### Data extraction

All the studies were selected according to the aforementioned inclusion criteria independently by two authors. Then the following information was extracted from each eligible study: first author's surname, publication year, country of origin, name of cohort, sample size, cases of BC, gender, age, smoking status, outcome measures, data on adjusted HRs/RRs and their 95% CIs, and covariates for adjustment in the multivariable model. Any discrepancies were resolved by discussion or by consulting a third author.

### Statistical analysis

Firstly, we transformed reported HRs/RRs and the corresponding 95% CIs to their logarithms and standard errors (SEs). Incidence density ratios and RRs were considered as HRs in meta-analyses (Ronksley et al., 2011; Zeng et al., 2016b). Then the heterogeneity across studies was explored using the *Q-*test (*P* ≤ 0.10 indicated statistical significance) and the I^2^ statistic (*I*^2^ ≥ 50% suggesting significant heterogeneity) (Zeng X. T. et al., 2015; Zeng et al., 2016a,b). In the presence of significant heterogeneity, a random-effects model was used; otherwise, the fixed-effect model was adopted. Subgroup analyses stratified by adjustment for smoking status or alcohol consumption, study design (prospective or retrospective), country of origin, and gender (male, female, or both) were also performed. HR was chosen as the pooled estimate and subgroup analysis was performed according to the reported estimation. Cumulative meta-analysis of studies was conducted in chronological order to assess the sequential contributions of studies published over time (Muellerleile and Mullen, 2006; Zeng X. T. et al., 2015; Zeng et al., 2016a,b). Besides, effects of follow-up duration, study design and confounder adjustment on overall findings were inspected by sensitivity analysis (Zeng et al., 2016b). If the number of included studies was sufficient (>9) (Egger et al., 1997), we used the funnel plot to investigate the underlying publication bias. All analyses were conducted using the inverse-variance model in RevMan version 5.3 software (Zeng et al., 2013).

## Results

### Characteristics of included studies

**Figure 1:**
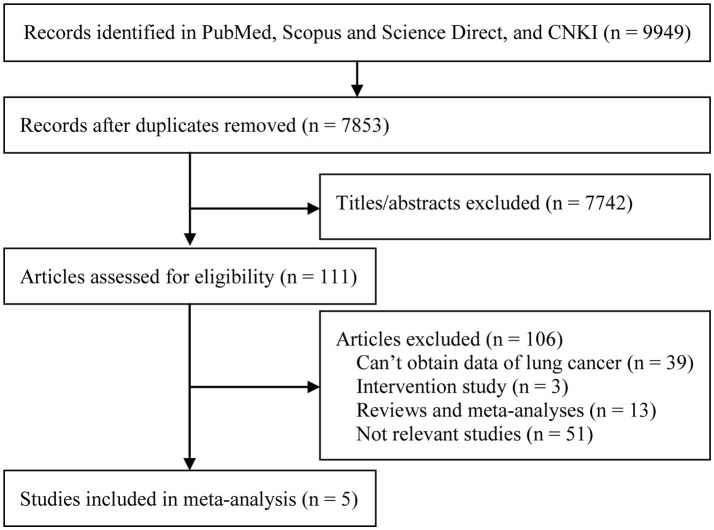
Study selection flow diagram.

Our initial search yielded a total of 9,949 citations. Of them, 7 records with fulltext were retrieved for further assessment. Then, two studies were excluded owing to insufficient data (Virtanen et al., 2014; Dizdar et al., 2017). As a result, five cohort studies containing 1,104 BC cases of 298,476 samples were eventually included in our meta-analysis (Michaud et al., 2008, 2016; Arora et al., 2010; Wen et al., 2014; Nwizu et al., 2017). Figure 1 presented the study selection process. Of the five included studies, four were prospective cohort studies (Michaud et al., 2008, 2016; Arora et al., 2010; Nwizu et al., 2017), and one was a retrospective cohort study (Wen et al., 2014). Countries of origin included USA(Michaud et al., 2008, 2016; Nwizu et al., 2017), Sweden (Arora et al., 2010), and China (Wen et al., 2014). All studies reported adjusted HRs with their 95%CIs. There were two studies concerning cross-over population (Michaud et al., 2008, 2016), but we included both of them because the latest one (Michaud et al., 2016) only focused on never smokers. The characteristics of included studies were described in Table 1, and the covariates for adjustment of each study were listed in Table 2. Of them, three studies (Michaud et al., 2008; Arora et al., 2010; Dizdar et al., 2017) adjusted smoking and alcohol, two studies (Michaud et al., 2008; Arora et al., 2010) adjusted smoking, alcohol, and diabetes.

**Table 1:** Characteristics of included cohort studies.

**Study (cohort/ country)**	**Study design**	**Age (years)**	**Gender (cases/samples)**	**Outcomes (assessment method)**	**Median follow-up (years)**	**HR (95% CI)**
(Michaud et al., 2008) (Health Professionals Follow-Up Study, HPFS/ USA)	Prospective	40–75	Male (543/48375)	Incident cancer (Medical records or pathology reports)	17.7	1.17(0.96-1.43)
(Arora et al., 2010) (The Swedish Twin Registry/ Sweden)	Prospective	38-77	Both (174/30666)	Incident cancer and cancer mortality (ICD)	27	1.13(0.59-2.20)
(Wen et al., 2014) (The National Health Insurance (NHI) program in Taiwan/ China)	Retrospective	≥20	Both (188/153566)	Incident cancer (ICD-9)	2	0.93(0.70-1.22)
			Female (54/71086)			1.26(0.74-2.16)
			Male (161/73810)			0.83(0.60-1.15)
(Michaud, 2016) (Health Professionals Follow-Up Study, HPFS/ USA)	Prospective	40-75	Male never smokers (222/19933)	Incident cancer (Medical records or pathology reports)	26	1.38(0.93-2.05)
(Nwizu et al., 2017) (Women's Health Initiative Observational Study (WHI-OS)/ USA)	Prospective	68.3 (mean)	Female (199/65869)	Incident cancer (ICD-O-2)	8.32 ± 3.95	1.10(0.81-1.49)

*CI, confidence interval; HR, hazard ratio; ICD, International Classification of Diseases; NA, not available*.

**Table 2:** Adjustments in studies included in the meta-analysis.

**Study**	**Adjustment**
Michaud et al., 2008	Age, race, physical activity, diabetes, alcohol, body mass index, geographical location, height, calcium intake, total calorific intake, red-meat intake, fruit and vegetable intake, vitamin D, and smoking
Arora et al., 2010	Gender, age, education, employment, number of siblings, smoking, smoking status of partner, alcohol, diabetes, and body mass index
Wen et al., 2014	Gender, age, diabetes, hypertension, and hyperlipidemia
Michaud et al., 2016	Age, race, alcohol use, physical activity, history of diabetes, body mass index, geographical location, height, and nonsteroidal anti-inflammatory drug use
Nwizu et al., 2017	Age, smoking status (pack-years), and body mass index

### Meta-analysis

**Figure 2:**
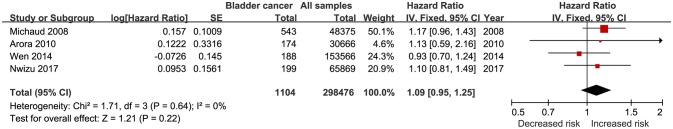
Forest plot of periodontal disease and risk of bladder cancer in overall population.

Four prospective cohort studies (Michaud et al., 2008, 2016; Arora et al., 2010; Nwizu et al., 2017) revealed a positive but non-significant association of PD with risk of BC, and pooled analysis of four studies (Michaud et al., 2008; Arora et al., 2010; Wen et al., 2014; Nwizu et al., 2017) by fixed-effect model also indicated no significant association between them (*HR* = 1.09, 95% *CI* = 0.95–1.25, *P* for test = 0.22; *I*^2^ = 0%, *P* for heterogeneity = 0.64, *Q* = 1.71; Figure 2). Cumulative meta-analysis by adding studies one-by-one in chronological order showed a similar result (Table 3). Considering that only four studies were included for main estimates, the publication bias assessment was not performed.

**Table 3:** Results of cumulative meta-analysis of studies in chronological order.

**Study**	**No. of studies (cases/samples)**	**Heterogeneity**		**Model**	**Meta-analysis**		
		***I*^2^(%)**	***p***		**HR**	**95% CI**	***p***
Michaud et al., 2008	1(543/48375)	NA	NA	NA	1.17	0.96-1.43	0.12
Arora et al., 2010	2(717/79041)	0	0.92	Fixed-effect	1.17	0.97-1.41	0.11
Wen et al., 2014	3(905/232607)	0	0.43	Fixed-effect	1.09	0.93-1.27	0.29
Nwizu et al., 2017	4(1104/298476)	0	0.64	Fixed-effect	1.09	0.95-1.25	0.22

*CI, confidence interval; HR, hazard ratio; NA, not available*.

### Subgroup and sensitivity analyses

Table 4 demonstrated the results of subgroup and sensitivity analyses. All the outcomes were similar to the overall result. Significant heterogeneity was only detected in the male subgroup (*I*^2^ = 68%, P for heterogeneity = 0.08), all other subgroups with no or non-significant homogeneity. The retrospective cohort, male and female, and Asia subgroups with *OR* < 1.00; all others were >1.00. Sensitivity analyses by omitting the study with longest follow-up duration, study with shortest follow-up duration, co-twin study, or the study without adjustment of smoking status, all indicated that the overall result was robust and with good homogeneity.

**Table 4:** Results of subgroup and sensitivity analyses.

**Subgroup analyses**	**No. of studies (cases/samples)**	**Heterogeneity**		**Model**	**Meta-analysis**		
		***I^2^*(%)**	***p***		**HR**	**95%CI**	***p***
**ADJUSTED COVARIATES**							
Smoking and alcohol	3(916/144939)	0	0.95	Fixed	1.15	0.98-1.35	0.09
Smoking, alcohol, and diabetes	2(717/79041)	0	0.92	Fixed	1.17	0.97-1.41	0.11
**SMOKING STATUS**							
None smokers	1(222/19933)	NA	NA	NA	1.38	0.93-2.05	0.11
**STUDY DESIGN**							
Prospective cohort	3(916/144939)	0	0.95	Fixed	1.15	0.98-1.35	0.09
Retrospective cohort	1(188/153566)	NA	NA	NA	0.93	0.70-1.24	0.62
**GENDER**							
Male and female	2(362/184232)	0	0.59	Fixed	0.96	0.74-1.24	0.76
Female	2(253/136955)	0	0.46	Fixed	1.14	0.87-1.48	0.34
Male	2(704/122185)	68	0.08	Random	1.01	0.72-1.41	0.95
**COUNTRY OF ORIGIN**							
Asia	1(188/153566)	NA	NA	NA	0.93	0.70-1.24	0.62
Europe	1(174/30666)	NA	NA	NA	1.13	0.59-2.16	0.71
USA	2(742/114244)	0	0.74	Fixed	1.15	0.79-1.36	0.10
**SENSITIVITY ANALYSES**							
Study with longest follow-up duration omitted	3(930/267810)	0	0.43	Fixed	1.09	0.94-1.26	0.25
Study with shortest follow-up duration omitted	3(916/144939)	0	0.95	Fixed	1.15	0.98-1.35	0.09
Co-twin study excluded	3(930/267810)	0	0.43	Fixed	1.09	0.94-1.26	0.25
Excluded study without adjustment of smoking status	3(916/144939)	0	0.95	Fixed	1.15	0.98-1.35	0.09

*CI, confidence interval; HR, hazard ratio; NA, not available*.

## Discussion

Growing evidence suggests that PD has common risk factors with a number of other non-communicable diseases and conditions, and the exploration of the common behavioral and environmental risk factors may contribute to the effective prevention of PD. In this meta-analysis, the relationship between PD and BC was evaluated by incorporating five cohort studies with 1,104 BC cases and 298,476 samples, and a positive but non-significant result was obtained. The cumulative, subgroup and sensitivity analyses all supported that this non-significant result was not influenced by type of study design, follow-up time, ethnicity, smoking and alcohol status, or gender.

Our results are similar to that of another meta-analysis on colorectal cancer, which indicated null associations between PD and specific types of cancers. However, evidence from other studies that reported positive association between PD and cancers with positive results indicating that PD might play an important role in the development of certain cancers (Sadighi Shamami and Amini, 2011; Zeng et al., 2013, 2016b; Yin et al., 2016; Maisonneuve et al., 2017; Michaud et al., 2017), which are not in agreement with the result of the present study. These findings might imply that the cancers from different locations had their own specificity, despite belonging to aerodigestive cancers (including cancers in oral cavity and pharynx, esophagus, stomach, pancreas, liver, colon, and rectum/anus) (Ansai et al., 2013). It is valuable to investigate whether bladder wall has a special structure that could resist the invasion of periodontal pathogens and their products.

It's biologically plausible that smoking and PD act jointly to increase the risk of cancer (Zeng et al., 2016b). To the best of our knowledge, smoking is the most important risk factor for BC accounting for approximately 50% of cases (Freedman et al., 2011), so we analyzed the results of studies adjusted for smoking status. In addition, the prospective cohort study by Michaud et al. was only implicated in the male never smokers (Michaud et al., 2016). All the results uncovered that the link between PD and BC was not influenced by smoking status. This was interesting and could not be explained by the etiology of BC. Thus, it's hypothesized that there's some sort of interaction between periodontal pathogens and smoking in the bladder which may reduce the effect of smoking or periodontal pathogens. What's more, the two diseases are both diseases of aging, and aged population are more likely to suffer from PD and BC, which may be the increasing influence of some age-associated factors aggravating the effect of PD and BC in the older, such as poor oral hygiene practice, hypotrophic absorption, decreased of defense and immunity capacity (Baelum and Lopez, 2013; Malats and Real, 2015); however, our studies failed to further explain and quantify the effect of age-dependent risk on the association of the two disease, for the limitation of a broad age ranking of included studies. Putative mechanisms involved in the association between PD and cancers included infection and inflammation, which had been proposed as the important risk factors (Sadighi Shamami and Amini, 2011; Michaud et al., 2017). Considering that bladder is filled with urine, the concentration of periodontal pathogens and/or their products in urine was diluted; besides, there might exist some inflammatory cytokines and/or factors in the urine which might react with periodontal pathogens and/or their products. All of these valuable points are needed to be further detected in the future.

Several limitations existed in observational studies on PD and cancer risk (Bueno et al., 2015), which became especially problematic in meta-analysis (Zeng X. et al., 2015). PD is extremely difficult to be measured and quantified in observational studies, because the assessment of PD requires several periodontal measurements, and clinical definitions may change over time. Currently, the clinical diagnosis of PD is based on the measurement of the pocket probing depth (PPD), community periodontal index of treatment need (CPITN), clinical attachment loss (CAL), bleeding on probing (BOP), and alveolar bone loss (ABL) with or without radiographic examinations (Bueno et al., 2015). The self-reported questionnaire is also commonly used in epidemiological investigations. Our meta-analysis included five cohort studies; there were two reporting assessment of PD using ABL (Michaud et al., 2008, 2016), two using self-reported questionnaire (Arora et al., 2010; Nwizu et al., 2017), and one that did not mention the procedures for assessment of PD (Wen et al., 2014), that might influence the precision of our results. Additionally, PD is a complex disease with multiple classifications that require a number of oral measurements, including CAL, PPD, and ABL. This disorder can manifest as a chronic or aggressive, generalized or localized progression in different degrees. All of these factors need to be considered in large-scale and multi-center studies. Furthermore, standardized measurements and definitions for categories in studies examining cancer outcomes should be applied, which allow meta-analysis with PD as an exposure and make the results more comparable and complete. Although this meta-analysis included one retrospective study from Taiwan (Wen et al., 2014), no studies conducted in mainland China or other Asian countries were identified. The genetic background may result in some differences in the same disease between Asians and Caucasians, or other ethnic populations; hence, more well-designed studies are required to further verify the results of current meta-analysis in the future.

In conclusion, this meta-analysis revealed that PD might not be associated with risk of BC, which was not affected by study design, duration of follow-up, ethnicity, smoking and alcohol status, or gender. However, we could not downplay this issue, because PD is implicated in the occurrence mechanisms of many systematic diseases, and BC patients with PD also need early aggressive treatment to prevent other diseases. Besides, further relevant studies should adopt standardized measurements and definitions for categories of PD, thereby making the results more comparable and complete.

## Author contributions

W-ZX, X-HW, and X-TZ designed this study. W-ZX and Y-HJ performed search and collected data. W-DL re-checked data. Y-HJ and X-TZ performed analysis. X-TZ wrote the manuscript. W-DL and X-HW reviewed the manuscript.

### Conflict of interest statement

The authors declare that the research was conducted in the absence of any commercial or financial relationships that could be construed as a potential conflict of interest.
